# Visual acuity in patients with non-functioning pituitary adenoma: Prognostic factors and long-term outcome after surgery

**DOI:** 10.1016/j.bas.2023.102667

**Published:** 2023-08-26

**Authors:** Erik Uvelius, Stig Valdemarsson, Johan Bengzon, Björn Hammar, Peter Siesjö

**Affiliations:** a Neurosurgery, Department of Clinical Sciences Lund, Lund University, Skåne University Hospital, Lund, 221 85, Lund, Sweden; b Department of Clinical Sciences, BMC F12, Lund University, 221 84, Lund, Sweden; c Ophthalmology, Department of Clinical Sciences Lund, Lund University, Skåne University Hospital, 221 85, Lund, Sweden

**Keywords:** Pituitary adenoma, Pituitary neoplasms, Transsphenoidal surgery, Visual function, Treatment outcome

## Abstract

**Background:**

Visual acuity (VA) and visual field defects (VF) are evaluated in the preoperative management of non-functioning pituitary adenoma (NFPA). The former is less studied than the latter.

**Research question:**

To analyze preoperative factors, including adenoma volumetry, associated with reduced VA and postoperative improvement of VA over five years after surgery.

**Methods:**

Eighty-seven patients who had primary surgery for NFPA were retrospectively reviewed. Eyes were categorized by best/worse preoperative VA. Ophthalmology review was performed before surgery, at three months, one to two years, and five years post-surgery.

**Results:**

Reduced VA in any eye was present in 55%. VA of the worse eye improved in 77% and normalized in 54%. The majority improved within three months. Additional cases with VA improvement were seen at 1–2 years after surgery. No further improvement was seen five years after surgery. Fifty percent of patients with, per definition, normal preoperative VA showed improved VA postoperatively. Tumor height above the sella in the sagittal plane was the best radiological predictor of reduced VA. Volumetry did not add to accuracy. Age, sagittal tumor height and visual field defects were risk factors of preoperative reduced VA. No predictors of postoperative recovery were identified.

**Conclusion:**

Half of patients with reduced VA recover fully. All patients, independent of age and degree of VA reduction, may improve. No predictors of recovery were found. Early improvement is common and improvement beyond two years is unlikely. The frequency of reduced VA is underestimated. The present results could be of value in pre- and postoperative counseling.

## Highlights

• In patients with NFPA, visual acuity (VA) is less well-studied than visual field defects.
• Half of patients will make a full recovery of VA after surgery.
• All patients have the potential for improvement, as no predictor of recovery was found.
• The ICD-10 criteria for normal VA likely underestimate the presence of impaired VA.
• The present results could be of aid in peri-operative patient counseling.

## 1 Background

Pituitary adenomas are common lesions accounting for 15% of all intracranial tumors (Mete and Lopes, 2017). Non -functioning pituitary adenomas (NFPA) are most prevalent, causing symptoms by impairing hormone secretion or by compressing surrounding neural structures (Tjornstrand et al., 2014; Ferrante et al., 2006) The anterior visual pathways are located in the suprasellar cisterns above sella turcica and the pituitary gland (Rhoton, 2002) and it is well accepted that visual field (VF) defects and reduced visual acuity (VA) occur by compression and dislocation of these structures (Miller et al., 2016). Reduced VA and VF defects constitute strong indicators for surgical treatment of NFPAs, thus ophthalmological examinations are extensively used in clinical surveillance of these tumors (Molitch, 2017). Even though technical innovations for the evaluation of both VA and VF defects, such as OCT and automated perimetry, have been introduced, routine assessment is still performed by Snellen charts and manual perimetry in many institutions. Current research has focused on patients with VF defects, exploring factors correlated to preoperative VF defects and postoperative improvement (Lithgow et al., 2019). Correlations between VF defects and tumor height, dislocation of the optic chiasm as well as tumor volume have been thoroughly analyzed (Schmalisch et al., 2012; Ikeda and Yoshimoto, 2009; Bonomo et al., 2020; Ogra et al., 2014; Eda et al., 2009; Gan et al., 2018), while the effect on VA has not been examined to the same extent. Considering the multifactorial pathophysiology of visual impairment caused by pituitary adenoma (Kerrison et al., 2000; Cottee et al., 2003) we hypothesized that VA, specifically representing the central visual field, and function of the anatomical center of the optic nerves (Miller et al., 2016), might be associated with specific cut-off values in predictors for preoperative deficits as well as postoperative improvement. The present retrospective study was primarily initiated to identify preoperative factors, including radiological measurements, associated with reduced VA and factors associated with postoperative improvement and normalization of VA. The overall aim was to extend knowledge on risk factors and outcome of reduced VA in NFPA patients and thereby aiding in preoperative counseling of patients with reduced VA due to NFPA.

## 2 Methods

### 2.1 Patients

**Fig. 1:**
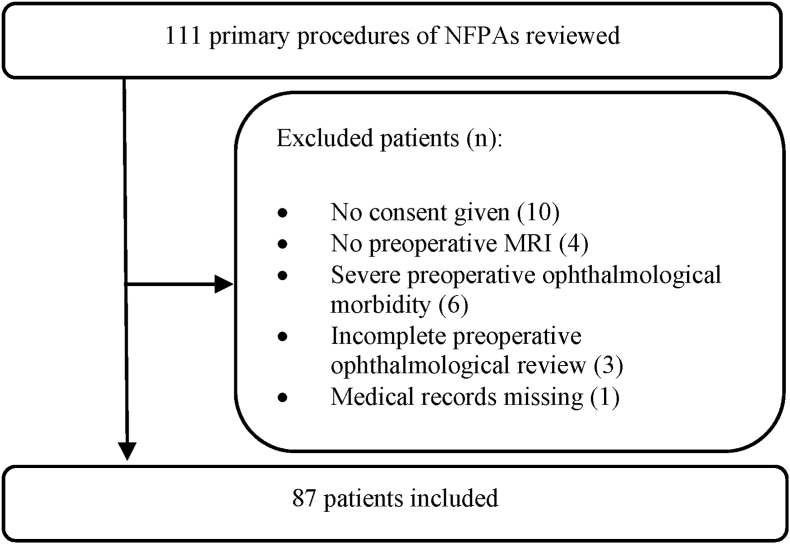
Flow chart presenting patient selection and exclusions.

Data were retrospectively collected from medical records of adult patients (≥18 years of age) with histologically confirmed NFPAs compressing (displacing, thinning, or reshaping) the optic apparatus, having undergone transsphenoidal surgery as primary treatment between May 2009 and June 2015. Reoperations were excluded to avoid confounders from previous surgery. The study period was selected to allow for five years of follow-up. The study was performed under ethical approval from the Regional Ethical Review Board in Lund, Sweden (Ref. No: 2012/374). During the study period, 111 patients fulfilled the inclusion criteria. After written consent was obtained from patients alive, 101 patients were reviewed. Further exclusions resulted in a study population of 87 individuals (Fig. 1). All procedures were performed with a fully endoscopic transsphenoidal technique.

### 2.2 Ophthalmological and radiological evaluations

Preoperative and postoperative ophthalmology review (at three months, one to two years, and five years after surgery) included best-corrected VA (Snellen chart); VF testing with either automated static perimetry with Humphrey Field Analyzer, alternatively Octopus perimetry, or with manual (kinetic) Goldmann perimetry (Table 1); slit lamp examination and fundoscopy were performed as well as extraocular motility testing. Normal VA was defined as Snellen decimal notation 0.8 or higher (Ophtalmology. Visual Standards, 2002). VA below normal was categorized into mild visual impairment (Snellen notation 0.7 to 0.3), moderate visual impairment (Snellen notation 0.3 to 0.1) and severe visual impairment (Snellen notation ≤0.1) according to ICD-10 (World Health Organization, 2019). A significant change was classified as two rows or more on the Snellen chart. Median deviation (MD) and Visual Field Index (VFI), both global indices of VF compared to the normal population, were noted in patients examined pre- and post-operatively with perimetry using the Humphrey Field Analyzer. In patients having perimetry by other methods, only interpretation by the ophthalmologist was recorded (graded as worsened, unchanged, improved, or normalized VF).

**Table 1:** Summary of VF defects according to preoperative ophthalmology review.

	Reduced VA (n = 48)	Unaffected VA (n = 39)
No VF defect, n (%)	2 (4)	14 (36)

VF defects, n (%)	46 (96)	25 (64)
Unilateral defect		
Superotemporal quadrantanopia, n (%)	0 (0)	3 (8)
Unilateral temporal hemianopia, n (%)	4 (8.5)	1 (2.5)
Other	0 (0)	0 (0)

Bilateral defects		
Superotemporal quadrantanopia, n (%)	10 (21)	6 (15.5)
Bitemporal hemianopia, n (%)	22 (46)	7 (18)
One eye temporal hemianopia, other eye superotemporal quadrantanopia, n (%)	4 (8.5)	1 (2.5)
One eye temporal hemianopia, other eye severe constriction, n (%)	3 (6)	2 (5)
Homonymous hemianopia, n (%)	1 (2)	0 (0)
Severe constriction, n (%)	0 (0)	1 (2.5)
Other combinations, n (%)	2 (4)	5 (13)

**Fig. 2:**
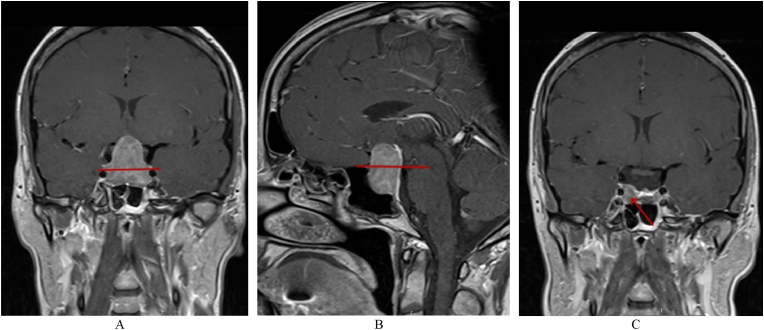
A and B illustrate the coronal and sagittal reference lines. Tumor volume and height above the red lines were separately analyzed in addition to total tumor volume and height in relation to pre-operative VA and postoperative VA recovery. Image C illustrates an adequately decompressed optic apparatus with no tumor remnant reaching the chiasm, even though residual tumor is visible (red arrow). (For interpretation of the references to colour in this figure legend, the reader is referred to the Web version of this article.)

All patients had a preoperative MRI scan from which tumor size and volume were extracted. Adenoma height was measured from the upper limit of the adenoma to a line drawn from planum sphenoidale to the top of the posterior clinoids in the sagittal plane; and to a line connecting the top of the intracavernous carotid arteries bilaterally in the coronal plane (Fig. 2), as described by Schmalisch et al. (2012). Adenoma volume and volumes above the aforementioned coronal and sagittal lines were determined with slice-by-slice volumetry with the computer software OsiriX (Rosset et al., 2004). A postoperative MRI, three months after surgery, determined if the optic apparatus was decompressed. The optic apparatus was considered decompressed if no tumor remnant reached the optic nerves, chiasm, or optic tract on the follow-up MRI (Fig. 2).

### 2.3 Statistical methods

Statistical analyses were performed with the free statistical software R (R Core Team, 2012). As NFPA affects the anterior visual pathways, data from each eye could not be considered independent. Consequently, eyes were categorized by best or worse preoperative VA and analyzed accordingly throughout the follow-up. Thus, patients with reduced VA in any eye were compared with patients with bilateral normal VA. Continuous variables are presented as mean ± standard deviation if not skewed, categorical variables as number (%), and finally ordinal or skewed continuous variables are presented as median (interquartile range). To determine differences between patients with and without reduced VA, *t*-test was used for continuous variables and Mann-Whitney test for skewed or ordinal data. Fischer’s exact test or Chi-squared test were used for dichotomous variables. Logistic multivariable regression determined pre-operative factors associated with preoperative VA and factors predicting improvement in VA after surgery. P-values <0.05 were considered significant. The following variables were included in the initial analysis: Age, gender, KNOSP-grade, successful decompression of the anterior visual pathways according to postoperative MRI, adenoma volumes and heights, and finally MD and VFI. Receiver operating characteristic (ROC) curves illustrated sensitivity and specificity of tumor heights and volumes in determining preoperatively reduced VA and postoperative recovery. The Youden index was used to determine the optimal cut-off value with highest sensitivity and specificity. Area under the ROC curves (ROC-AUC) with 95% confidence interval were calculated to determine the predictive power of the analysis. AUC values between 0.7 and 0.9 indicate moderate accuracy whereas AUC values over 0.9 indicate high accuracy.

## 3 Results

### 3.1 Patients

The mean age of the 87 patients included was 62 ± 14 years, with 31 patients (36%) being females. Reduced VA, in one or both eyes, was present in 48 patients (55%), and VF defects in 71 patients (82%). Bitemporal VF defects were common while unilateral defects occurred in less than 10% of patients (Table 1). Patients presenting with reduced VA were older than patients with normal VA (65 years and 58 years, respectively, p = 0.01). Postoperative MRI confirmed adequate decompression of the optic apparatus in 93% of patients.

Postoperative deterioration of VA was seen in 5 patients (6%). All cases were asymmetrical with reduction occurring in only one eye. Out of these patients, two had a preoperative reduction of VA, and both regained preoperative VA levels one year after surgery. Three patients with normal preoperative VA experienced VA reduction after surgery, out of these, two had regained full VA one year after surgery, leaving only one patient (1.1%) with a permanent postoperative reduction in VA in one eye.

### 3.2 Visual acuity at presentation

Amongst patients with affected VA (n = 48), the median VA was 0.7 (range 0.3 to 1.3) in the best eye and 0.4 (range from light perception to 0.7) in the worse affected eye. Twelve patients (25%) presented with moderate VA impairment (Snellen notation 0.3 to 0.1) and ten patients (21%) presented with severe impairment (Snellen notation ≤0.1). There was a notable asymmetry in VA as no patient demonstrated severe VA impairment in both eyes (best eye range: 0.4 to 1.0) and only two patients had moderate bilateral impairment.

### 3.3 Radiological correlation between adenoma size and reduced VA

**Fig. 3:**
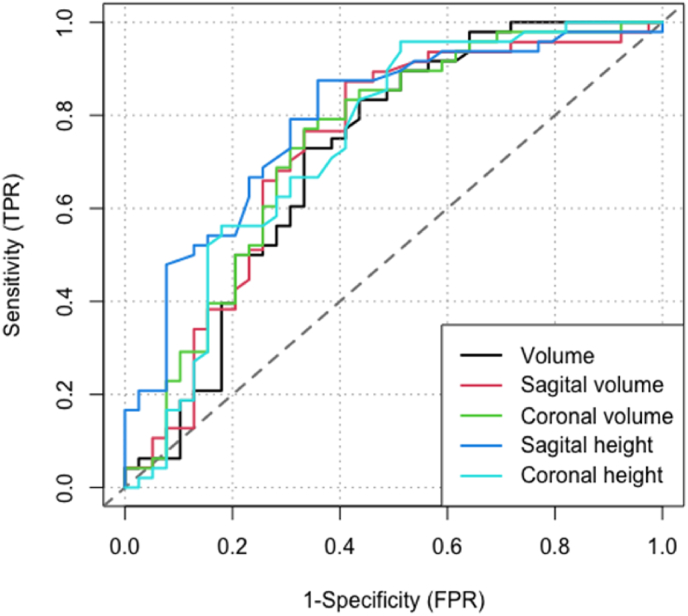
ROC-curves illustrating sensitivity and specificity of tumor measurements on preoperative MRI in predicting preoperative reduction in VA.

The median tumor volume was significantly larger in patients with reduced VA, 8.5 (5.5 to 10.6) cm^3^ compared to 4.4 (3.2 to 8.3) cm^3^ in patients with and without reduced VA, respectively, p<0.001. Additionally, the median volume above the sagittal line was 2.7 (1.9 to 5.1) cm^3^ in patients with reduced VA and 1.1 (0.7 to 2.4) cm^3^ in patients with normal VA (p<0.001). The mean height of tumor growth above the sagittal line was 13.5 ± 4.4 mm and 9.2 ± 3.3 mm in patients with and without affected VA (p<0.001). Similar results were seen comparing the volume above the coronal reference line and coronal tumor height. The radiological measurements on preoperative MRI are presented in Table 2. ROC curves illustrating the sensitivity and specificity of aforementioned radiological measurements in predicting preoperatively reduced VA are presented in Fig. 3. The best test accuracy, determined by the highest ROC-AUC, was seen with tumor height above the sagittal line, AUC 0.79 (95% CI: 0.69 to 0.89), cut-off: 10 mm, sensitivity 0.88 and specificity 0.64) indicating a moderate test accuracy. In general, the different measurements of volumes and tumor heights were equally accurate. There were no significant differences between any of the curves in a pairwise analysis of ROC-AUCs. In attempting to identify a cut-off value predicting postoperative improvement, only low AUCs, sensitivities and specificities were observed, indicating low test accuracy for the prediction of VA recovery.

**Table 2:** Summary of radiological measurements on preoperative MRI.

	Reduced VA (n = 48)	Unaffected VA (n = 39)	P-value
Adenoma volume, cm3, median (IQR)	8.5 (5.5–10.6)	4.4 (3.2–8.3)	<0.001
Adenoma volume above coronal line^a^, cm^3^, median (IQR)	4.5 (3.3–6.7)	2.8 (1.9–4.1)	<0.001
Adenoma volume above sagittal line^b^, cm^3^, median (IQR)	2.7 (1.9–5.1)	1.1 (0.7–2.7)	<0.001

Vertical adenoma height, coronal^a^, mm, median (IQR)	18.2 (15.4–20.7)	14.0 (12.0–17.6)	<0.001
Vertical adenoma height, sagital^b^, mm, median (IQR)	13.5 (10.9–15.5)	9.2 (6.9–11.3)	<0.001

a Measurement above a line connecting the top of the intracavernous carotid arteries bilaterally in the coronal plane.

b Measurement above a line drawn from planum sphenoidale to the top of the posterior clinoids in the sagittal plane.

### 3.4 Preoperative factors associated with reduced VA

In patients with reduced VA, the median MD and VFI were both significantly lower, indicating more extensive VF defects (Table 3). This could also be seen in logistic regression models when adjusting for multiple factors in the best and worse eye separately. Furthermore, regressions showed that increasing tumor height in the sagittal plane (OR 1.04, p = 0.04), increasing age (OR 1.04, p = 0.003), and low MD of the worse eye in patients having had preoperative perimetry with the Humphrey field analyzer (OR 0.98, p = 0.03), were significant factors associated with reduced VA prior to surgery.

**Table 3:** VF indices in patients with and without reduced preoperative VA.

	Reduced VA, best eye	Unaffected VA, best eye	p-value	Reduced VA, worse eye	Unaffected VA, worse eye	p-value
**MD, median, dB(IQR)**	-5.9 (-13.9 to 2.2)	-1.3 (-2.6 to -0.2)	<0.001	-9.3 (-20.1 to -5.0)	-1.8 (-3.9 to -0.2)	<0.001
**VFI, median, %(IQR)**	84 (55.5 to 95.25)	98 (95 to 99)	<0.001	72.5 (43.25 to 84.25)	96 (87 to 99)	<0.001

### 3.5 Postoperative improvement in patients with reduced VA

Thirteen patients with preoperative reduction in VA were lost to first follow-up.

**Fig. 4:**
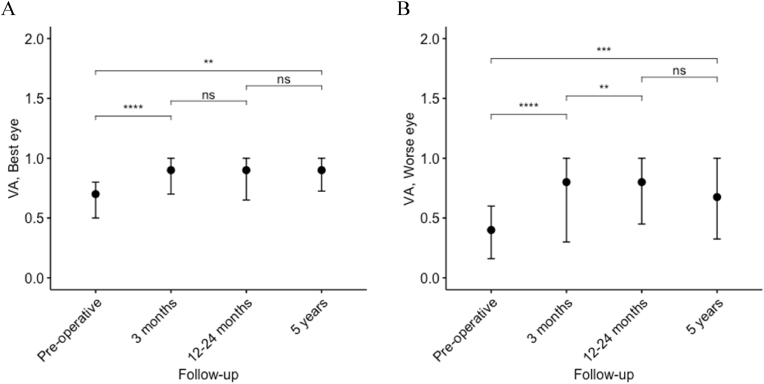
Change in VA during follow-up in best and worse eye separately. **A**, Va of the best eye, median (IQR). **B**, VA of the worse eye, median (IQR). *p<0.05,**p<0.01,***p<0.001,****p<0.0001.

In reviewing the eye with the best VA, at three-month follow-up, improved VA was seen in 17/35 patients (49%), of which a majority, 15/35 patients (43%), had regained normal VA. Two additional patients showed significant improvement at the next follow-up one to two years after surgery, and three patients showed further improvement compared to the three-month follow-up. Thus, in total, 19/35 patients (54%) improved in VA of the best eye post-surgery. Median VA improved from 0.7 (0.5–0.8) preoperatively to 0.9 (0.65–1.0) one to two years after surgery (p<0.001). No further improvement was seen at the five-year follow-up. Changes in median VA during follow-up are presented in Fig. 4.

The VA of the eye with the lowest preoperative VA showed improvement in 25/35 patients (71%) three months after surgery, and 19/35 patients (54%) of these had fully restored VA. Only a minority, a further two patients, showed new improvement at next follow-up, one to two years after surgery, although eight patients showed additionally better VA compared to the first follow-up. In total 27/35 patients (77%) improved in VA of the worse eye. The median VA of the worse eye improved from 0.4 (0.16–0.6) preoperatively to 0.8 (0.45–1.0) one to two years after surgery (p<0.001, Fig. 4). No further improvement of VA at five years follow-up was observed. Normalization of VA was seen with all levels of reduced preoperative VA, though more common in patients with mild preoperative impairment (50%) compared to patients with moderate (25%) or severe impairment (30%).

Regression analysis did not show any factor significantly predicting improvement. The analyses included decompression of the optic apparatus dichotomized as yes or no. There was no significant statistical difference in postoperative recovery of VA between completely decompressed as compared to non-decompressed optic pathways. Having one eye with normal VA did not affect the likelihood of improvement of the affected eye. Age, grouped by quartiles, did not indicate that increasing age negatively influenced the likelihood of postoperative VA recovery. Improvement in the worse eye at follow-up one to two years post-surgery was seen as follows: 18–52 years: 80% (n = 4/5), 53–63 years: 57% (n = 4/7), 64–72 years: 69% (n = 9/13), and finally age above 73 years: 80% (n = 8/10), p = 0.74.

### 3.6 Postoperative improvement in VF in relation to VA

Improvement of VF, when preoperatively affected, was seen in 48/54 patients (89%). In patients with and without reduced VA, improvement at first follow-up, three months post-surgery, was seen in 31/36 patients (86%) and 17/18 patients (94%), respectively (p = 0.65). Further improvement at follow-up one to two years after surgery only occurred in patients with reduced preoperative VA. The overall rate of full VF recovery was 31%, seen in 21% and 50% with and without reduced preoperative VA, respectively (p = 0.01).

### 3.7 Outcome in patients defined as having normal preoperative VA

Twenty out of 39 patients (51%), considered to have no VA impairment according to the ICD-10 criteria, showed improvement in VA during follow-up. Median VA of the worse eye, in this subgroup, increased from 0.8 (0.8–1.0) prior to surgery to 1.0 (0.8–1.0) one to two years post-surgery, though statistical significance was not reached (p = 0.08). The majority, 15 patients, improved prior to the three-month follow-up.

## 4 Discussion

The present retrospective study was initiated to extend current knowledge regarding the effect NFPA specifically has on VA and what result can be expected by surgical intervention. In our series of 87 patients with surgically treated NFPA, preoperative impairment of VA and VF was found in 55% and 82% respectively. Specifically, decreased VA was seen more often among older patients and was also related to sagittal tumor height and severity of VF defects. Most importantly, improvement in VA after surgery was noted in the best and worse seeing eyes in 54% and 77%, respectively. Improvement was most often seen among those with mild VA impairment but was also noted among those with moderate and even severe preoperative reduction in VA. Recovery of VA was seen over all ages, also among the oldest patients. Overall, most of the recovery was observed early, at three months postoperatively, without obvious further improvement at follow-up one to two years and five years after surgery. Post-operative deterioration of VA was uncommon and often followed by complete recovery in most cases. Thus, in principle, each individual patient can be informed that there is a chance of improved VA as no obvious factors predict outcome and that the risk of postoperative VA decline is small, about 1%.

Predicting outcome is of great importance in aiding preoperative decision-making and patient counseling. Patient age, symptom duration, preoperative VA and VF, surgeons experience, grade of resection, and finally tumor size have all, inconsistently, been associated with surgical outcome [ (Ogra et al., 2014), (Kerrison et al., 2000), (Monteiro et al., 2010; Muskens et al., 2017; Gnanalingham, 2005; Anik et al., 2018; Barzaghi et al., 2011; Chabot et al., 2015; Juraschka et al., 2014). Our results are in accordance with a 2017 meta-analysis reporting that 14–84% of patients with NFPA present with reduced VA and 28–100% with VF defects (Muskens et al., 2017). Similar to our findings, the aforementioned meta-analysis showed a pooled VA improvement rate of 67.5% (Muskens et al., 2017). Additionally, in our material, we note full normalization of VA in 54% of cases when considering the ICD-10 definition of normal VA being a Snellen decimal notation 0.8 or above (World Health Organization, 2019). The fact that complete decompression of the optic apparatus as a variable predicting postoperative recovery of VA did not reach statistical significance likely illustrates the multifactorial pathophysiology of visual disturbance due to optical compression. Thus, patients can be informed that VA, when affected, will improve in a majority of cases, though the chance of complete recovery is around 50%.

Notably, half of our patients considered to have unaffected VA according to the ICD-10 definition, still showed improvement beyond the level of normal VA. Population-based evaluations of VA show Snellen decimal 0.97 being the median VA for 70–82 year old’s while the mean VA for younger adults varies around 1.3 (Sjöstrand et al., 2011). This would suggest Snellen decimal 0.8 being a crude estimation of normal VA that does not exclude affected VA and thus might underestimate the true prevalence of VA deficits in patients with NFPA. Thus, VA, when deemed unaffected by NFPA still improve after surgery in 50% of cases.

The pathophysiology of visual decline caused by pituitary adenoma is multifactorial (Cottee et al., 2003). Distension of nervous tissue in experimental spinal cord research, has been correlated with ischemia and demyelination (Sun et al., 2012). Adding to this is the anatomical weakness of the blood supply of the central parts of the chiasm (Bergland and Ray, 1969). Considering these facts, one might theorize that an adenoma with a larger suprasellar volume, and thereby larger contact surface to the chiasm, could cause increased tension in the axons of the anterior visual pathways. If so, suprasellar tumor volume could be expected to show a higher correlation with VA than just the suprasellar tumor height. However, our present results could not confirm this hypothesis. In fact, volumetric measurements, resulting in ROC-AUC between 0.73 and 0.74, did not add to the predictive power of linear radiological measurements of sagittal and coronal tumor height. Additionally, suprasellar extension in the sagittal plane was the only tumor measurement found to be a significant risk factor for preoperative reduction in VA. The present results indicate that adenomas reaching 10 mm above the sagittal reference line may be predictive of preoperative reduction in VA with a sensitivity of 88% and a specificity of 64%. Thus, patients can be informed that VA is at risk if the tumor extends 10 mm or above the reference line, which should be conveyed to patients hesitant about undertaking surgery.

It has been suggested that the improvement of visual impairment after decompression of the anterior visual pathways evolves in two or three phases. An early phase, seen immediately after surgery to one-week post-surgery, likely caused by the removal of conduction block caused by ischemia and reduced axoplasmic transport, is followed by a second phase one month to four months post-surgery explained by remyelination (Kerrison et al., 2000; Clifford-Jones et al., 1985). Finally, further improvement seen six months up to three years post-surgery is less often described or explained (Kerrison et al., 2000). These phases have been demonstrated in clinical practice regarding VF defect (Kerrison et al., 2000; Gnanalingham, 2005) where the majority of improvement, as well as all cases of complete recovery, was seen during the initial six months after surgery (Kerrison et al., 2000; Gnanalingham, 2005). No similar improvement phases have been reported regarding VA restitution. Dekkers et al. (2007) explored the recovery of VA up until one year after surgery for NFPA, noting a mean VA increase in both eyes and that about half of the patients showed continuing improvement between three and 12 months. Our present results confirm an early phase of VA recovery and extend previous knowledge (Dekkers et al., 2007), by our longer follow-up period, indicating a possible improvement of VA in some patients as late as one to two years after surgery (Fig. 4). However, we did not find any further improvement at five-year follow-up. Thus, patients can be informed that VA might improve up until one to two years after surgery though not beyond this time frame.

Although we did find increasing age to be associated with reduced VA prior to surgery, we did not find age to be predictive of VA improvement following surgery as VA recovery was seen also among our oldest patients. In contrast, in a recent paper, patient age was the only significant predictor of VA improvement following transsphenoidal surgery as younger age correlated to better prognosis (Butenschoen et al., 2021). The significant negative correlation between increasing age and VA improvement following surgery in that study, might, as the authors point out, be explained by coexisting ophthalmologic comorbidities. In our series, patients with severe ophthalmological comorbidity (n = 6) were excluded. Thus, elderly patients can be informed that VA, when affected, might improve with surgery despite patients' age, although other factors might impede the improvement.

## 5 Conclusions

VA improves and recovers in a similar manner as previously reported regarding VF defects after surgery of NFPA. Patients with reduced VA should be identified and receive active treatment early as VA improve in only 54% and 77% of the best and worse seeing eye, respectively. However, only about half of these patients will have a chance of full VA recovery. All patients, independent of age and degree of VA reduction, have the potential for improvement, as we found no factor predictive of recovery. Improvement will likely occur during the first months after surgery, but VA can, unlike VF, in some cases improve up to one to two years after surgery. The level of visual acuity at this point is likely permanent. Finally, applying the ICD-10 criteria for normal VA, Snellen notation above 0.8, might underestimate the presence of impaired VA in patients with NFPA. The data presented could likely be of aid in pre- and post-operative patient counseling.

## Funding

This research did not receive any specific grant from funding agencies in the public, commercial, or not-for-profit sectors.

## Informed consent

Informed consent was obtained from all individual participants included in the study.

## Contributions

All authors have made substantial contributions to the present article. Conceptualization and design (EU, SV, BH, PS). Acquisition of data (EU, BH). Analysis and interpretation of data (EU, SV, JB, BH, PS). Drafting the article (EU) Text revision (EU, SV, JB, BH, PS). Final approval of the version submitted (EU, SV, JB, BH, PS)

## Declaration of competing interest

The authors declare that they have no known competing financial interests or personal relationships that could have appeared to influence the work reported in this paper.
